# Obstructive sleep apnea -related hypertension: a review of the literature and clinical management strategy

**DOI:** 10.1038/s41440-024-01852-y

**Published:** 2024-08-29

**Authors:** Kazuki Shiina

**Affiliations:** Department of Cardiology, Tokyo Medical University, Tokyo, Japan

**Keywords:** Obstructive sleep apnea, Continuous positive airway pressure (CPAP), Hypertension, Antihypertensive medication, Vascular remodeling.

## Abstract

Obstructive Sleep Apnea (OSA) and hypertension have a high rate of co-occurrence, with OSA being a causative factor for hypertension. Sympathetic activity due to intermittent hypoxia and/or fragmented sleep is the most important mechanisms triggering the elevation in blood pressure in OSA. OSA-related hypertension is characterized by resistant hypertension, nocturnal hypertension, abnormal blood pressure variability, and vascular remodeling. In particular, the prevalence of OSA is high in patients with resistant hypertension, and the mechanism proposed includes vascular remodeling due to the exacerbation of arterial stiffness by OSA. Continuous positive airway pressure therapy is effective at lowering blood pressure, however, the magnitude of the decrease in blood pressure is relatively modest, therefore, patients often need to also take antihypertensive medications to achieve optimal blood pressure control. Antihypertensive medications targeting sympathetic pathways or the renin-angiotensin-aldosterone system have theoretical potential in OSA-related hypertension, Therefore, beta-blockers and renin-angiotensin system inhibitors may be effective in the management of OSA-related hypertension, but current evidence is limited. The characteristics of OSA-related hypertension, such as nocturnal hypertension and obesity-related hypertension, suggests potential for angiotensin receptor-neprilysin inhibitor (ARNI), sodium-glucose cotransporter 2 inhibitors (SGLT2i) and glucose-dependent insulinotropic polypeptide receptor/ glucagon-like peptide-1 receptor agonist (GIP/GLP-1 RA). Recently, OSA has been considered to be caused not only by upper airway anatomy but also by several non-anatomic mechanisms, such as responsiveness of the upper airway response, ventilatory control instability, and reduced sleep arousal threshold. Elucidating the phenotypic mechanisms of OSA may potentially advance more personalized hypertension treatment strategies in the future.

**Figure:**
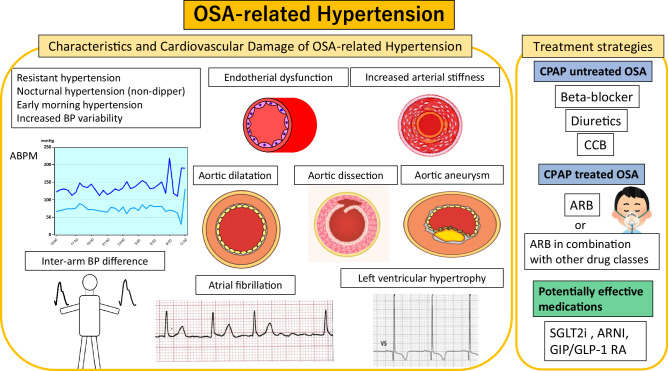
Clinical characteristics and management strategy of OSA-related hypertension. OSA obstructive sleep apnea, BP blood pressure, ABPM ambulatory blood pressure monitoring, CPAP continuous positive airway pressure, LVH left ventricular hypertrophy, ARB: angiotensin II receptor blocker, SGLT2i Sodium-glucose cotransporter 2 inhibitors, ARNI angiotensin receptor-neprilysin inhibitor, CCB calcium channel blocker, GIP/GLP-1 RA glucose-dependent insulinotropic polypeptide receptor and glucagon-like peptide-1 receptor agonist.

Clinical characteristics and management strategy of OSA-related hypertension. OSA obstructive sleep apnea, BP blood pressure, ABPM ambulatory blood pressure monitoring, CPAP continuous positive airway pressure, LVH left ventricular hypertrophy, ARB: angiotensin II receptor blocker, SGLT2i Sodium-glucose cotransporter 2 inhibitors, ARNI angiotensin receptor-neprilysin inhibitor, CCB calcium channel blocker, GIP/GLP-1 RA glucose-dependent insulinotropic polypeptide receptor and glucagon-like peptide-1 receptor agonist.

## Abstract (web summary)

Clinical characteristics and management strategy of OSA-related hypertension. OSA: obstructive sleep apnea, BP: blood pressure, ABPM: ambulatory blood pressure monitoring, CPAP: continuous positive airway pressure, LVH: left ventricular hypertrophy, ARB: angiotensin II receptor blocker, SGLT2i: Sodium-glucose cotransporter 2 inhibitors, ARNI: angiotensin receptor-neprilysin inhibitor, CCB: calcium channel blocker, GIP/GLP-1 RA: glucose-dependent insulinotropic polypeptide receptor and glucagon-like peptide-1 receptor agonist.

## Introduction

Obstructive sleep apnea (OSA) is characterized by recurrent episodes of complete or partial collapse of the upper airway during sleep, resulting in apnea or hypopnea, and is recognized as an independent risk factor for cardiovascular disease such as hypertension, heart failure, arrhythmia, and coronary heart disease [1, 2]. OSA increases blood pressure (BP) by enhancing various risk factors including the sympathetic nervous system, renin-angiotensin-aldosterone system (RAAS), and inflammation through mechanisms such as obesity, decreased intrathoracic pressure, pulmonary stretch receptor stimulation, chemoreceptor stimulation, hypoxemia, and hypercapnia [3, 4]. The 2023 European Society of Hypertension (ESH) Guidelines for the diagnosis and treatment of hypertension emphasize the importance of the obese state and of the metabolic syndrome, one of the causes of hypertension, as the main partially reversible risk factors for OSA [5].

The presence of OSA has been related to an increase in the prevalence and incidence of hypertension, regardless of other factors (OR: 1.5–2.9) [6, 7]. In fact, approximately 30–50% of hypertensive patients have OSA, whereas 50% of patients with OSA present hypertension [6], especially about 80% of patients with resistant hypertension have OSA. Therefore, screening for OSA is recommended in patients with resistant hypertension [8, 9].

This review aims to summarize the latest findings on the clinical features of OSA-related hypertension to its treatment strategies.

## Symptoms and clinical findings in OSA

In patients with OSA, extremely loud snoring and sleep apnea are typical symptoms, often prompting visits based on the partner’s observations (Table 1). However, the rational clinical examination systematic review by Myers, et al. [10] reported that the most useful observation for identifying patients with OSA was nocturnal choking or gasping (summary likelihood ratio [LR], 3.3; 95% CI, 2.1–4.6) when the diagnosis was established by apnea-hypopnea index: AHI ≥ 10/h). Snoring is common in OSA patients but is not useful for establishing the diagnosis (summary LR, 1.1; 95% CI, 1.0–1.1). Daytime excessive sleepiness is a common subjective clinical symptom, although it has been reported that awareness of symptoms is often lacking [11], particularly among patients with cardiovascular diseases [12, 13]. As there is controversy regarding the association of morning headache and obstructive sleep apnea syndrome (OSAS), Goksan, et al. demonstrated that prevalence of morning headache increases with increasing OSAS severity [14]. Atrial stretch due to the large negative pressure swings by OSA results in secretion of atrial natriuretic peptide, causing nocturia. The prevalence of OSA increases with factors such as obesity and aging, but in Asia, there are many non-obese OSA patients related to craniofacial bony restriction [15].

**Table 1:** Symptoms and clinical finding of OSA

Symptoms	Snoring
	Witnessed apneas by sleep partner
	Excessive daytime sleepiness
	Morning headaches
	Difficulty concentrating
	Nocturia
	Insomnia with repeated awakenings
	Erectile dysfunction
	Depression
Clinical finding	Obesity
	Long face
	Hypertrophy of the soft palate, tonsils, and tongue
	Macroglossia
	Atrial fibrillation
	Left ventricular hypertrophy
	Chronic kidney disease

Therefore, when examining hypertensive patients, it is important to pay attention to typical symptoms such as sleepiness and abnormalities in facial and pharyngeal morphology, even in non-obese patients, and to actively perform OSA screening tests when these abnormalities are suspected. Additionally, it is necessary to actively suspect OSA in patients with left ventricular hypertrophy (LVH), aortic disease, atrial fibrillation (AF), and those undergoing dialysis [9].

## New concept of mechanisms in OSA

Recently, OSA has been considered to be caused not only by upper airway anatomy but also by several non-anatomic mechanisms [16]. These factors include the responsiveness of the upper airway responce, ventilatory control instability [17], and reduced sleep arousal threshold [18]. The relative contributions of these processes may vary from one patient to another and have therapeutic implications [19]. For example, upper-airway stimulation device is a new treatment for OSA that targets the responsiveness of the upper airway response [20]. Future treatments for OSA-related hypertension may need to consider these phenotypes.

## Hypertension Risk and OSA

OSA and hypertension are not merely comorbidities; OSA itself can potentially be a causative factor for hypertension. The Wisconsin Sleep Cohort study, a prospective, community-based study, it has been demonstrated that an increase in AHI independently of age and body mass index (BMI) is associated with the new-onset hypertension [6]. In contrast, the 5-year follow-up study of the Sleep Heart Health Study, conducted with 2470 participants without hypertension at admission, found that after adjusting for BMI, AHI was no longer a significant predictor of hypertension [21]. The findings that do not support the relationship between OSA and hypertension were attributed to the lower rate of participants with moderate to severe OSA. Indeed, the vast majority of the participants included in the 5-year follow-up of the Sleep Heart Health Study had mild OSA [21]. On the other hand, Marin, et al. demonstrated that the presence of OSA was associated with increased adjusted risk of incident hypertension in a large prospective cohort study (median follow-up periods 12.2 years) without hypertension [22]. The incidence of hypertension increased with severity of OSA. These findings suggest that untreated “severe” OSA is independently of BMI associated with an increased risk for developing new-onset hypertension, and there is a “dose–response” relation between OSA and the risk of developing hypertension.

## Pathogenesis of Hypertension in OSA

The mechanisms promoting hypertension in OSA are multifactorial and complex. Sympathetic activity due to intermittent hypoxia and/or fragmented sleep is the most important mechanisms triggering the elevation in BP in OSA. The pathophysiology begins with obstructed airfow into the lungs, which causes transient hypoxia and hypercapnia. The sympathetic nervous system is activated simultaneously by these repetitive blood gas derangements, which stimulate both central and peripheral chemoreceptors, apnea-induced cessation of pulmonary stretch receptor-mediated inhibition of central sympathetic outflow, and silencing of sympathoinhibitory input from carotid sinus baroreceptors by reductions in stroke volume and BP during obstructive apneas. When the apnea is interrupted by arousal from sleep, the latter process simultaneously augments sympathetic nervous activity and reduces cardiac vagal activity [3, 4, 23]. The result is a postapneic surge in BP [24].

These acute adverse effects of OSA on the autonomic nervous system are not confined to sleep. Patients with OSA and cardiac dysfunction also have elevated sympathetic nervous activity and depressed cardiac vagal activity when awake [23]. The mechanisms for such daytime carryover effects remain unclear but may relate to the adaptation of chemoreceptor reflexes or central processes governing autonomic outflow.

Consequently, RAAS is activated, the endothelin-1 level is increased, and the nitric oxide level is decreased, all of which contribute to the increase in vascular resistance and the development of hypertension [25, 26]. Sympathetic hyperactivity leads to a proinflammatory state, resulting in endothelial dysfunction and increased arterial stiffness [27–29].

## Characteristics of OSA-related HT

### Resistant Hypertension

**Fig. 1:**
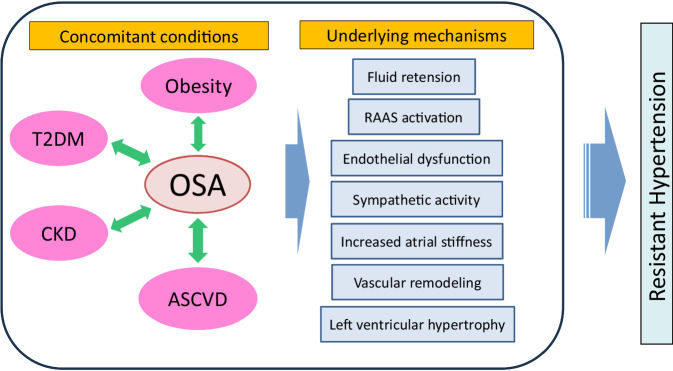
Proposed pathways through which OSA may contribute to the development of resistant hypertension. OSA obstructive sleep apnea, RAAS RAAS: renin–angiotensin– aldosterone system, T2DM type 2 diabetes mellitus, CKD chronic kidney disease, ASCVD atherosclerotic cardiovascular disease

Resistant hypertension is defined as BP that is uncontrolled despite using ≥3 medications of different classes (Table 2), commonly a long-acting calcium channel blocker (CCB), angiotensin converting enzyme inhibitor (ACEI) or angiotensin II receptor blocker (ARB) and a diuretic (Fig. 1). Multiple studies have demonstrated a high prevalence of OSA in patients with resistant hypertension. The prevalence is reported to be 70–80% [30–34]. Several mechanisms may exlain the potential role of OSA in promoting resistant hypertension [35]. These include sympathetic nervous system activation, endothelial dysfunction, increased arterial stiffness, and fluid retention due to OSA. Among these mechanisms, increased arterial stiffness due to OSA is a major cause of resistant hypertension. Roderjan et al. reported that among resistant hypertensives, the more severe the apnea was associated with the greater the arterial stiffness, and that patients with increased pulse wave velocity (PWV) have an adverse clinical and polysomnographic profile pointing to a higher cardiovascular risk, especially women, patients with true resistant hypertension [36]. We have demonstrated that OSA and metabolic syndrome were independently associated with elevated PWV in large Sleep Cohort [29]. Although it is not clear what the roles of arterial stiffness in contributing to resistant hypertension are, it is reasonable to speculate that the vascular remodeling promoted by OSA may exacerbate BP in patients with hypertension [37].

**Table 2:** Characteristics of OSA-related HT

Resistant hypertension
Nocturnal hypertension (non-dipper/riser type)
Early morning hypertension
Masked hypertension
Left ventricular hypertrophy・Left ventricular diastolic dysfunction
Increased blood pressure variability (short-term, long-term)
Increased arterial stiffness
Inter-arm blood pressure difference

### Non-dipper phenomenon

OSA-related hypertension is predominantly nocturnal and characterized by a non-dipping pattern [38–40]. Systolic BP (SBP) and diastolic BP (DBP) reduce by ~10 mmHg (about 10–20%) during sleep, but this dipping phenomenon is reversed in those with OSA. The prevalence of non-dipping was 84% in a population of untreated patients with mild to severe OSA [41]. OSA increases sympathetic nerve activity due to arousals in sleep, which counteracts the normal nocturnal BP dip and results in increased intravascular pressure. This chronic hypertension leads to vascular remodeling, decreased endothelial production of vasodilatory nitric oxide, and insensitive baroreceptors, further inhibiting the reflex of nocturnal BP dip [28, 42, 43]. In patients with severe OSA, positive airway pressure (CPAP) turns a non-dipping into a dipping BP profile [44].

### BP variability (BPV)

In OSA, BP variability (BPV) has been studied mainly as very short-term (beat-to-beat) and short-term (24-hour BP profile) variability [45, 46].

BP measured on consecutive heartbeats has been demonstrated to be highly variable, due to repeated peaks during sleep, so that an accurate assessment of nocturnal BP levels in OSA may require peculiar methodologies [47–50].

Consistent evidence indicates that the presence of OSA may be associated with increased short-term BPV, but the information on its relationship with long-term BPV, assessed on a visit-to-visit variability (VVV) is limited [51]. We observed that patients with severe OSA had significantly higher systolic VVV than controls matched for age, BMI and SBP [52]. Moreover, in this study, the plasma noradrenaline level and the AHI were independently and positively correlated with VVV and VVV was significantly reduced by CPAP. In a different study, Kansui et al. demonstrated the impact of OSA on long-term (yearly) BPV in Japanese work-site population [53].

### Inter-arm BP difference

Inter-arm SBP difference (IAD) is a non-invasively and easily measurable parameter. Recent evidence suggests the existence of correlations between IAD and the risk of cardiovascular events and mortality in patients with hypertension, diabetes mellitus, and coronary artery disease, as also in the general population [54]. IAD of BP is important but the measurement methodology has a major influence on IAD results. According to a meta-analysis, the number of subjects with a systolic and diastolic IAD ≥ 10 mmHg was significantly lower when BP measurements were performed simultaneously instead of sequentially [55]. This could have overestimated the prevalence of IAD ≥ 10 mmHg. The results from Tokyo Sleep Heart Study, moderate to severe OSA was independently associated with the IAD accessed by simultaneously BP measurements [56]. The plausible explanation is that the negative intrathoracic pressure caused by OSA may exert an adverse impact on the structural properties of the thoracic aorta.

## Cardiovascular damage by OSA-related HT

### Cardiac morphology and function

#### Left Ventricular Hypertrophy (LVH)

Several studies have observed an association between OSA and LVH [57–59], but it has been difficult to demonstrate an association between OSA and higher LVH independent of obesity and hypertension. Indeed, Usui, et al. demonstrated that no significant differences in left ventricular mass index by OSA severity in 74 healthy non-obese men [60]. However, recent meta-analysis showed that OSA was significantly associated with an increased risk of LVH (OR = 1.70, 95% CI 1.44–2.00, P < 0.001) [61]. Although significant variability in prevalence estimates exists between studies, recent meta-analysis suggests that in the OSA setting concentric LVH is more frequent than eccentric LVH [62].

#### Left ventricular systolic function

Literature reports concerning left ventricular systolic function in OSA patients are controversial. The meta-analysis by Yu, et al. demonstrated that significant decreases in left ventricular ejection fraction (LVEF) were observed in OSAS patients [63], however, the alterations in LVEF seemed not to be remarkable enough to induce obvious clinical symptoms of LV dysfunction. Recent study demonstrated that global longitudinal strain (GLS), a more sensitive measurement of LV systolic function, is impaired in patients with OSA, thus allowing to identify subclinical alterations of the systolic function not captured by LVEF [64].

#### Left ventricular diastolic function

Several studies demonstrated the association between OSA and　echocardiographic parameters of left ventricular diastolic dysfunction. Wachter, et al. reported that moderate-to-severe OSA is independently associated with diastolic dysfunction in a primary care cohort of 352 patients with cardiovascular risk factors [65]. OSA may be independently associated with left ventricular diastolic dysfunction perhaps due to higher LV mass [66]. Usui, et al. reported that coexistence of OSA and metabolic syndrome is independently associated with LVH and diastolic dysfunction in Japanese sleep cohort [67]. Clinicians should pay attention to the significance of the coexistence of these disorders so as to prevent the development of heart failure with preserved LVEF.

Based on these results, although comorbidities such as hypertension play a role in OSA, it is particularly associated with LVH and decreased left ventricular diastolic function. Therefore, it is important to consider the presence of OSA in patients with hypertension that exhibits these characteristics.

### Atrial fibrillation (AF)

The prevalence of OSA in patients with atrial fibrillation (AF) is extremely high [68, 69], making screening for OSA essential in these patients. The high-frequency intermittent hypoxia, negative intrathoracic pressure, atrial stretching, neurohumoral activation, and chronic concomitant conditions, such as hypertension, metabolic syndrome, and obesity, associated with OSA create progressive structural remodeling of the atrium [69]. This progressive atrial structural remodeling, along with the electrophysiological changes contributes to the reentry mechanism for AF and establishes an arrhythmogenic substrate in the atrium.

Recently, we reported that nutritional status and sleep quality are associated with AF in patients with OSA [70]. Undernutrition, as assessed by the CONtrolling NUTritional status (CONUT) score [71], and reduced slow-wave sleep were factors significantly related to the presence of AF. The CONUT scores were calculated from total peripheral lymphocyte counts, the serum albumin levels, and total cholesterol levels. On the other hand, several meta-analyses have demonstrated that CPAP therapy [72, 73] suppresses the recurrence of pulmonary vein isolation for AF. Therefore, CPAP therapy should also be actively considered in managing BP and preventing AF recurrence in OSA-related hypertension with AF.

### Vascular remodeling

**Fig. 2:**
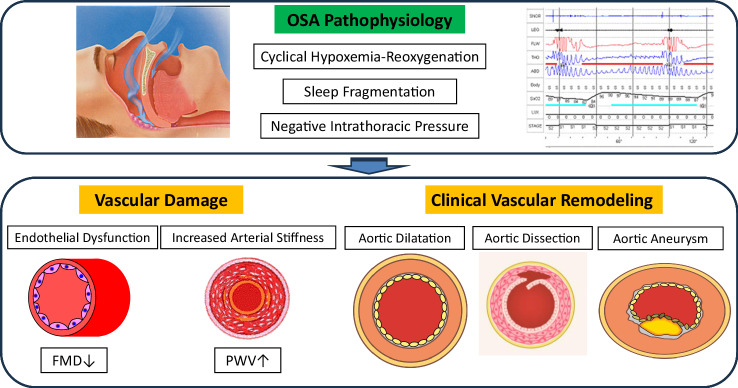
Vascular Damage by OSA. OSA: obstructive sleep apnea, FMD Flow mediated dilation, PWV pulse wave velocity

A potential pathophysiological role linking OSA to vascular remodeling (i.e., progressive aortic dilatation, increased risk for aneurysms, and aortic dissection) has been reported by several clinical studies [74–83]. Pathophysiological conditions associated with the development of these vascular remodeling in OSA include negative intrathoracic pressure, increased BP via sympathetic hyperactivity, and oxidative stress via cyclical hypoxemia-reoxygenation due to OSA (Fig. 2). Recent meta-analysis actually showed that aortic size was higher in patients with OSA than in their counterparts without OSA [75]. However, the results of this meta-analysis should be considered in the context of some limitations, such as the paucity of available data, and the methodological differences of the various studies.

Regarding the relationship between aortic dissection (AD) and OSA severity, a greater relation was found between moderate-to-severe OSA and AD (OR 4.43; 95% CI 2.59–7.59) [79]. Gaisl, et al. demonstrated the strong evidence for a positive association of thoracic aortic aneurysms (TAA) expansion with AHI [80]. On the other hand, in abdominal aortic aneurysms (AAA) patients, the rate of aortic diameter enlargement was significantly higher by 2.2 mm/year in the population with an AHI ≥ 30/h compared with an AHI 0–5/h [81]. We also demonstrated that patients with TAA, AAA, and AD showed high incidences of moderate to severe OSA [82]. Negative intrathoracic pressure could theoretically dilate the thoracic aorta via increased stress in the aortic wall, but would have little effect on the abdominal aorta. However, it is inconclusive which of the thoracic and abdominal vasculatures OSA more strongly impacts.

## Treatment of OSA-related hypertension

Among the treatment modalities that come to the fore in OSA-related hypertension are CPAP, antihypertensive medications (beta-blocker, diuretics, ARB and CCB), and renal denervation (RDN). There are currently no specific clinical recommendations on whether to prioritize CPAP or antihypertensive medications in OSA-related hypertension. However, in hypertensive patients with moderate to severe OSA accompanied by sleepiness, it is common to prioritize CPAP therapy to improve sleep quality. Weight loss, physical exercise, reducing alcohol consumption, and smoking cessation are among the primary lifestyle changes recommended for OSA-related hypertension [83].

### CPAP therapy

**Fig. 3:**
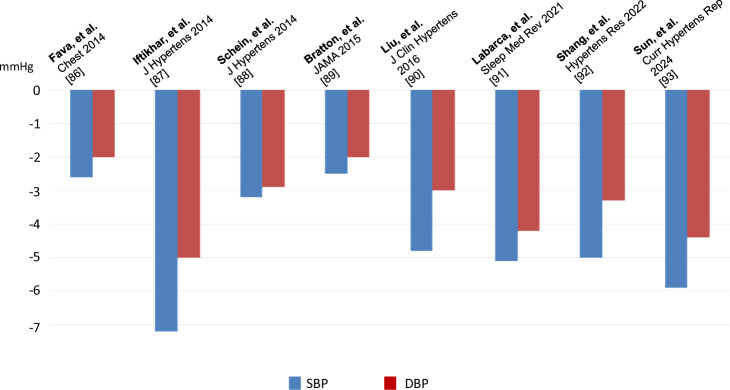
Recent meta-analyses regarding the effect of CPAP treatment on blood pressure. CPAP Continuous positive airway pressure, SBP Systolic blood pressure, DBP Diastolic blood pressure

A number of studies have demonstrated that CPAP has modest but significant BP-lowering effects of 2–7 mmHg in SBP and of 2–5 mmHg in DBP in OSA-related hypertension [84–91] (Fig. 3). The effect of CPAP on BP varies among patients (Fig. 3). Higher BMI, severe OSA (AHI ≥ 30), hypersomnolence, higher BP values, untreated hypertension, nocturnal hypertension, treatment-resistant hypertension and adherence to CPAP are variables that have been associated with a greater improvement in BP in several studies [92–97]. HIPARCO RCT found a significant correlation between CPAP usage and reductions in 24-h mean BP, SBP, and DBP [98]. Best results for quality of life improvements and optimal reductions in BP occur when CPAP usage exceeds 4 hour/night [87, 99]. Furthermore, recent meta-analyses suggest an even higher degree of daily CPAP adherence (at least 4.0–5.5 hour/night) to improve BP in patients with resistant hypertension and sleepiness [100].

In the patients with non-sleepy OSA, CPAP therapy have no overall beneficial effects on subjective sleepiness, SBP, or cardiovascular risk compared with no active therapy. OSA patients who were less sleepy had lower BMI and lower CPAP adherence. This probably might be due to a lower respiratory arousal threshold. Comprehensive management including an active lifestyle and regular support of CPAP use is key to managing this kind of OSA [101]. Furthermore, CPAP withdrawal results in a clinically relevant increase in BP (office SBP): +5.4 mm Hg, home SBP : +9.0 mm Hg), which is considerably higher than in conventional CPAP trials [102].

In patients with nocturnal hypertension (non-dipper/riser types), CPAP often selectively lowers BP during sleep, leading to a normal dipper pattern [103]. In the aforementioned studies, HIPARCO RCT, among patients with OSA and resistant hypertension, CPAP treatment for 12 weeks compared with control resulted in a decrease in 24 h mean (−3.1 mmHg) and DBP (−3.2 mmHg) and an improvement in the nocturnal BP pattern [98].

As mentioned above, the antihypertensive effects of CPAP are modest. However, CPAP therapy exert beneficial effects on sympathovagal balance and arterial stiffness, independent of BP lowering [104]. Therefore, patients with moderate-to- severe OSA-related hypertension should undergo CPAP therapy as a first-line treatment.

### Antihypertensive medications

CPAP therapy is effective at lowering BP, however, the magnitude of the decrease in BP is relatively modest, therefore, patients often need to also take antihypertensive medications to achieve optimal BP control (Table 3). However, current guidelines do not specify what type of antihypertensive therapy should be offered to patients with OSA and concomitant hypertension [5, 8]. An earlier study conducted by Kraiczi et al. compared the effects of atenolol, hydrochlorothiazide, amlodipine, enalapril, and losartan on office and ambulatory BP in 40 OSA-related hypertension patients [105]. Compared with the other four medications, atenolol lowered the office DBP as well as mean night-time ambulatory SBP and DBP. These findings support the hypothesis that overactivity of the sympathetic nervous system is the most important mechanism involved in the development of hypertension in patients with OSA. Kario, et al. reported the BP-lowering effects of CCBs and beta-blockers using a trigger sleep BP monitor with an oxygen-triggered function in OSA-related hypertension [106]. The BP-lowering effects of nifedipine on the mean and minimum sleep SBP were stronger than those of carvedilol, but sleep SBP surge was only significantly reduced by carvedilol.

**Table 3:** Studies that investigated the efficacy of various classes of antihypertensive medications for lowering blood pressure in patients with OSA

Author	Year	*n*	Study Design	Antihypertensives; dosage	CPAP (Y/N)	BP outcome
Kraiczi et al. [105]	2000	40	RCT; double blinded; balanced incomplete block design	Atenolol 50 mg Amlodipine 5 mg Enalapril 20 mg Hydrochlorothiazide 25 mg Losartan 50 mg	No	Atenolol reduced mean nighttime ambulatory SBP and DBP more effectively than did amlodipine, enalapril, or losartan (but not hydrochlorothiazide)
Pepin, et al. [107]	2010	23	RCT; crossover	Valsartan 160 mg	Yes	24 h mean BP: −2.1 mmHg (CPAP) vs −9.1 mmHg (Valsartan)
Kario, et al. [106]	2014	11	RCT; single center; crossover	Nifedipine 40 mg Carvedilol 20 mg	No	The BP-lowering effects of Nifedipine on the mean and minimum sleep SBPs were stronger than those of Carvedilol
Kasai, et. al. [110]	2014	16	Uncontrolled open trial; single center	Metolazone 2.5–5.0 mg Spironolactone 25–50 mg	No	Intensified diuretic therapy significantly reduced home morning SBP, DBP, nighttime DBP, and clinic SBP
Yang et al. [109]	2016	30	RCT; open trial; blank-controlled; single-center	Spironolactone 20–40 mg	No	Spironolactone reduced clinic and 24 h SBP and DBP, compared with control
Svedmyr, et al. [112]	2021	5970	Retrospective multicenter; cohort study	Monotherapy: β-blockers, Diuretics, CCB, ACEI/ARB, Centrally-acting drugs Combination therapy: combination of two drugs of different classes	No	β-blockers alone orβ-blockers in combination with a diuretic was associated with the lowest systolic pressure
Svedmyr, et al. [113]	2023	mono:1283/dual: 652	Retrospective multicenter; cohort study		Yes	ACEI/ARB, alone or in combination with other drug classes, provides a particularly strong reduction of BP and better BP control

*RCT* Randomized controlled trial, *CPAP* Continuous positive airway pressure, *ABPM* Ambulatory blood pressure monitoring, *BP* Blood pressure, *SBP* Systolic blood pressure, *DBP* Diastolic blood pressure, *CCB* Calcium channel blocker, *ACEI* Angiotensin-converting enzyme inhibitor, *ARB* Angiotensin II receptor blocker

On the other hand, in terms of suppressing organ damage, RAAS inhibitors, such as ARB, may be useful in patients with OSA-related hypertension, especially in obese patients, because the RAAS is hyperactive and LVH is a common complication [107, 108].

Fluid retension from the lower extremities to the upper body during sleep is strongly associated with OSA in hypertensive patients. Therefore, in OSA patients with obesity and a fluid retention, diuretics may be beneficial. Spironolactone reduced the severity of OSA and reduced BP in resistant hypertension patients with moderate-to-severe OSA [109, 110]. A propensity score-matched cohort analysis of data from the French national sleep apnea registry demonstrated that diuretics appear to have a positive impact on OSA severity in overweight or moderately obese patients with hypertension [111].

Recently, Svedmyr, et al. investigated 5970 hypertensive patients with OSA on current antihypertensive treatment from the European Sleep Apnea Database (ESADA) cohort [112]. Monotherapy with beta-blocker was associated with lower SBP, particularly in non-obese middle-aged males with hypertension. Conversely, the combination of a beta-blocker and a diuretic was associated with lower SBP and DBP in hypertensive patients with moderate–severe OSA. Furthermore, another report in ESADA cohort suggests that ACEI or ARB, alone or in combination with other drug classes, provides a particularly strong reduction of BP and better BP control when combined with CPAP in OSA [113]. Considering that CPAP will remove repetitive hypoxia, most arousals, and the chronic sympathetic activation, it is likely that other mechanisms, such as RAAS activation, may play a dominant role following OSA treatment. This is speculated to be the reason why ACEI or ARB were effective in the CPAP treated OSA.

### Sodium-glucose cotransporter 2 inhibitors (SGLT2i)

A recent series of mega-scale clinical trials for sodium-glucose cotransporter 2 inhibitor (SGLT2i) indicated cardio-renal protective effects of SGLT2i [114–118], and some SGLT2is have now become the first-line treatment for T2DM with comorbid atherosclerotic cardiovascular disease (ASCVD) and heart failure. Furthermore, several studies have reported a lowering effect of SGLT2i on BP [119, 120]. Although mechanisms underlying the BP-lowering effects of SGLT2i are unclear, SGLT2i presumably acts primarily by decreasing circulating plasma volume through osmotic and natriuretic diuresis in the early stages of administration and later by suppressing sympathetic nerve activity in the long term [121, 122]. Wojeck, et al. reported that Ertugliflozin reduced incident OSA [123]. In the meta-analysis, Lin, et al. demonstrated that SGLT2i was shown to reduce AHI [124]. These results suggest that SGLT2i may not only have beneficial effects on OSA-related hypertension but also on OSA itself [125].

### Angiotensin receptor-neprilysin inhibitor (ARNI)

The angiotensin receptor neprilysin inhibitor (ARNI) has recently been approved in Japan to treat hypertension [126]. Reductions in 24-hour, daytime, and nighttime BP have been documented during treatment with ARNI in patients with hypertension [127–129]. This potent 24-hour BP-lowering effects of ARNI may be effective for OSA-related hypertension characterized by resistant, nocturnal, and non-dipper hypertension [130]. Additionally, as previously mentioned, since OSA-related hypertension is associated with LVH [57–59, 61, 62] and left ventricular diastolic dysfunction [65–67], ARNI, which is characterized by so-called “reverse remodeling”, may be useful for OSA-related hypertension. Furthermore, in chronic heart failure patients with sleep apnea, ARNI treatment for 3 months in patients with OSA decreased the severity of OSA itself (the ENTRESTO-SAS study) [131].

However, both ARNI and SGLT2i are used in the United States to treat heart failure. In addition, there may be considerably less research on antihypertensive in OSA. Future research is needed to investigate the effect of ARNI and SGLT2i on BP in patients with OSA-related hypertension.

### Glucose-dependent insulinotropic polypeptide receptor/ glucagon-like peptide-1 receptor agonist (GIP/GLP-1 RA)

Recently, a study evaluating the safety and efficacy of tirzepatide for the treatment of OSA and obesity was published (The SURMOUNT-OSA trials) [132]. Tirzepatide is a long-acting glucose-dependent insulinotropic polypeptide (GIP) receptor and glucagon-like peptide-1 (GLP-1) receptor agonist that selectively binds to and activates both the GIP and GLP-1 receptors. The SURMOUNT-OSA trials were two 52-week, phase 3, multicenter, parallel-group, double-blind, randomized, controlled trials that were conducted at 60 sites across nine countries to evaluate the efficacy and safety of the maximum tolerated dose of weekly tirzepatide (10 mg or 15 mg) in adults with moderate-to-severe OSA and obesity. In this trial, tirzepatide reduced the AHI, body weight, hypoxic burden, high-sensitivity C-reactive protein concentration, and SBP [Estimated treatment differences :−7.6 mmHg (95% CI, −10.5 to −4.8), *P* < 0.001, not receiving CPAP group]. The effect of tirzepatide on OSA-related hypertension is expected in the future.

### Renal denervation

Increased sympathetic activity, consistently evident in patients with OSA, plays a key role in the development of resistant hypertension [35]. Therefore, OSA-related hypertension may represent a promising indication for RDN. In an RCT conducted with moderate-to-severe OSA patients with resistant hypertension, Warchol-Celinska, et al. demonstrated that RDN safely provided significant BP reduction compared with the control group [133]. However, the effect of RDN for OSA-related hypertension remains unclear due to differences in research design and other factors, such as sham procedure, ablations catheter, treatment adherence, sample size, observational periods, etc. Further large scale studies are warranted to assess the impact of RDN on OSA and its relation to BP decline and cardiovascular risk.

## Future directions

As previously mentioned, it has become clear that OSA is caused not only by upper airway anatomic factors but also by several non-anatomic mechanisms [16–19]. Therefore, it is hypothesized that the pathophysiology of OSA-related hypertension is also not a single condition but is divided into several phenotypes. Elucidating the phenotypic mechanisms of OSA may potentially advance more personalized hypertension treatment strategies in the future.

## Conclusions

OSA occurs at a high prevalence in hypertensive patients, particularly those with resistant hypertension. Additionally, it is highly prevalent among AF patients, warranting OSA screening. OSA-related hypertension is characterized by morning hypertension, nocturnal hypertension, non-dipper pattern, increased BPV, and pronounced arterial remodeling. CPAP therapy is the gold standard therapy for OSA but its effects on BP are relatively modest, often requiring combination therapy with antihypertensive medications. While there is insufficient evidence regarding the choice of antihypertensive medications, beta-blockers, diuretics, and ARBs may be used as monotherapy or in combination therapy depending on individual cases. Further evaluation of the efficacy of novel agents such as SGLT2i and ARNI, and GIP/GLP-1 RA is necessary. Elucidating the phenotypic mechanisms of OSA may potentially advance more personalized hypertension treatment strategies in the future.
